# Whole Transcriptome Analysis Revealed a Stress Response to Deep Underground Environment Conditions in Chinese Hamster V79 Lung Fibroblast Cells

**DOI:** 10.3389/fgene.2021.698046

**Published:** 2021-09-16

**Authors:** Liju Duan, Hongying Jiang, Jifeng Liu, Yilin Liu, Tengfei Ma, Yike Xie, Ling Wang, Juan Cheng, Jian Zou, Jiang Wu, Shixi Liu, Mingzhong Gao, Weimin Li, Heping Xie

**Affiliations:** ^1^Wangjiang Hospital, Sichuan University, Chengdu, China; ^2^Department of Rehabilitation Medicine Center, West China Hospital, Sichuan University, Chengdu, China; ^3^Department of Otolaryngology Head and Neck Surgery, West China Hospital, Sichuan University, Chengdu, China; ^4^Deep Underground Space Medical Center, West China Hospital, Sichuan University, Chengdu, China; ^5^Department of Ophthalmology, West China Hospital, Sichuan University, Chengdu, China; ^6^College of Water Resources & Hydropower, Sichuan University, Chengdu, China; ^7^Institute of Deep Earth Science and Green Energy, Shenzhen University, Shenzhen, China

**Keywords:** deep underground environment, V79 cells, mRNA, lncRNA, circRNA

## Abstract

**Background:** Prior studies have shown that the proliferation of V79 lung fibroblast cells could be inhibited by low background radiation (LBR) in deep underground laboratory (DUGL). In the current study, we revealed further molecular changes by performing whole transcriptome analysis on the expression profiles of long non-coding RNA (lncRNA), messenger RNA (mRNA), circular RNA (circRNA) and microRNA (miRNA) in V79 cells cultured for two days in a DUGL.

**Methods:** Whole transcriptome analysis including lncRNA, mRNAs, circ RNA and miRNA was performed in V79 cells cultured for two days in DUGL and above ground laboratory (AGL), respectively. The differentially expressed (DE) lncRNA, mRNA, circRNA, and miRNA in V79 cells were identified by the comparison between DUGL and AGL groups. Quantitative real-time polymerase chain reaction(qRT-PCR)was conducted to verify the selected RNA sequencings. Then, Gene Ontology (GO) and Kyoto Encyclopedia of Genes and Genomes (KEGG) pathway was analyzed for the DE mRNAs which enabled to predict target genes of lncRNA and host genes of circRNA.

**Results:** With |log_2_(Fold-change)| ≥ 1.0 and *p* < 0.05, a total of 1257 mRNAs (353 mRNAs up-regulated, 904 mRNAs down-regulated), 866 lncRNAs (145 lncRNAs up-regulated, 721 lncRNAs down-regulated), and 474 circRNAs (247 circRNAs up-regulated, 227 circRNAs down-regulated) were significantly altered between the two groups. There was no significant difference in miRNA between the two groups. The altered RNA profiles were mainly discovered in lncRNAs, mRNAs and circRNAs. DE RNAs were involved in many pathways including ECM-RI, PI3K-Akt signaling, RNA transport and the cell cycle under the LBR stress of the deep underground environment.

**Conclusion:** Taken together, these results suggest that the LBR in the DUGL could induce transcriptional repression, thus reducing metabolic process and reprogramming the overall gene expression profile in V79 cells.

## Introduction

An increasing number of countries have begun to develop deep Earth in order to cope with the space and resources of surface Earth being gradually consumed in the future (Xie et al., 2018). As China, exploring deep underground space and resources has become a national priority since 2016 (Xie et al., 2017), which shown that an increasing number of people could live and/or work in the underground space in the near future (Ranjith et al., 2017; Liu et al., 2018). Currently in South Africa, gold miners are able to work 4000 m underground (Ranjith et al., 2017). However, little is still known about the biological effects of the deep underground environment (Liu et al., 2018). The limited knowledge may result from the shortage of deep underground laboratories (DUGLs),such as the Gran Sasso National Laboratory (LNGS) in Italy, and the Waste Isolation Pilot Plant (WIPP) in the United States. Researchers who have historically focused on the growth of cultures in DUGLs have observed some interesting changes(e.g., cell growth delay, enzyme activity change and sensitivity to genetic damage factors) in living organisms exposed to low levels of radiation (Belli, 2017; Castillo and Smith, 2017; Liu et al., 2018). However, with limited publications it is challenging to know the reliability of the results (Castillo and Smith, 2017).

To understand the biological effects of the deep underground environment on humans and service for the development of deep underground space and resources, a DUGL in a cave with a rocky cover of 1470 m was set up in our previous research in Erdaogou Mine, with Jiapigou Minerals Limited Corporation of China National Gold Group Corporation (CJEM) (Liu et al., 2018). Similar to the 4000 m water equivalent (WE) of LNGS, the flux of the cosmic rays in the DUGL of CJEM could be considered negligible compared to the cosmic rays at the above ground surface (Liu et al., 2020). The radon concentration in the DUGL of CJEM was 3.7–5.5 pCi/L, which is slightly higher than LNGS (Liu et al., 2020). However, the total gamma (γ) radiation dose rate of terrestrial radiation was 0.03–0.05 μSv/h,which was consistent with the levels at LNGS (Liu et al., 2020). Meanwhile, the relative humidity was approximately same as LNGS at 99%. Except for oxygen (O_2_), the concentration of carbon dioxide (CO_2_) and air pressure of the DUGL were slightly higher than an above ground laboratory (AGL) (Liu et al., 2020).

Prior series studies have investigated biological changes of Chinese hamster V79 cells in LNGS (Antonelli et al., 2000; Satta et al., 2002; Carbone et al., 2009; Fratini et al., 2015). Published research also demonstrated that V79 cells were able to be cultured in the DUGL of CJEM and the proliferation of these V79 cells could be inhibited by low background radiation (LBR) in this deep underground environment (Liu et al., 2018). Furthermore, our prior study found that V79 cells cultured in the DUGL of CJEM presented an altered protein profile related to the ribosome, RNA transport, translation, energy metabolism, and DNA repair (Liu et al., 2020). Recent evidence revealed that non-coding RNAs participate in modulating numerous biological functions by regulating gene expression at transcriptional and post-transcriptional levels (Beermann et al., 2016). In the current study, we revealed further molecular changes by performing whole transcriptome analysis on the expression profiles of long non-coding RNA (lncRNA), messenger RNA (mRNA), circular RNA (circRNA) and microRNA (miRNA) in V79 cells cultured for two days in a DUGL. These data will be helpful to further understand the biological effects of the deep underground environment.

## Materials and Methods

### Cell Culture

The detailed cell culture methods are described in our previous research (Liu et al., 2020). Briefly, frozen Chinese hamster V79 lung fibroblast cells were purchased from Shanghai Enzyme-linked Biotechnology (China) and cultured in Dulbecco’s modified eagle medium (DMEM; Gibco, United States) with 10% fetal calf serum (Gemini, United States), and 50 U dm^–3^ penicillin and streptomycin (Gibco, United States). At more than 80% confluency, cells were passaged and divided into four T 25 flasks, and two of them were assigned to either the DUGL or AGL of CJEM, respectively. All cells were maintained in incubators at 37°C with 5% CO_2_. One flask of each location was used for growth curve and morphology analysis in previous researched (Liu et al., 2020), the another flask was cultured for passage. After passaging and two days culture, three flasks with enough cells for further analysis from each location were collected for the following experiments.

### RNA Preparation and Quality Control

After the cells were cultured for two days in either the AGL or DUGL, the total RNA per sample was extracted using Trizol reagent (Invitrogen, NY, United States) and was then used for RNA sequencing. The purity and integrity of RNA were examined by 1% agarose gel electrophoresis (Sigma-Aldrich,United States). Subsequently, further RNA integrity was verified using the Agilent 2100 Bioanalyzer (Agilent Technologies, CA, United States), The RNA integrity number (RIN) of all samples were more than 7.0, which were considered to meet the sequencing requirement. The RNA quantity was measured using the NanoDrop-2000 (NanoDrop Technologies, DE, United States). The ribosomal RNA (rRNA) was removed with the Ribo-Zero GoldKits (Epicentre, WI, United States).

### lncRNA and mRNA Sequencing and Data Processing

RNA (3 μg) from each sample was used for cDNA library construction (NEB Next Ultra Directional RNA LibraryPrep Kit for Illumina, Ispawich, United States). After the removal of rRNA (Ribo-Zero GoldKits), the rRNA-depleted RNAs were fragmented and used as templates to construct the cDNA library. First strand cDNA was synthesized using random hexamer primers. Next, second strand cDNA synthesis was performed using DNA polymerase I and RNase H. Libraries were amplified by polymerase chain reaction (PCR).

NovaSeq 6000 Illumina sequencing system (Illumina, San Diego, CA, United States) was used for RNA sequencing. The sequencing data was analyzed using CASAVA software for base calling. Raw data were transformed into FASTQ stored documents. To obtain clear reads for further analysis, reads containing adapters and ploy-N as well as low-quality reads were removed from the raw data. HiSAT2 software^1^ was used to align sequencing data according to the reference hamster genome [GCA_003668045.1 (Rupp et al., 2018), GenBank assembly accession].

The Coding-Non-Coding Index (CNCI), Coding Potential Calculator (CPC), Pfamscan, and Coding Potential Assessment Tool (CPAT) were used to analyze the coding potential of transcripts. The assembled transcripts without coding potential of their overlap became the candidate set of lncRNAs.

The read count for each gene in each sample was counted by HTSeq, and Fragments Per Kilobase of transcript, per Million mapped reads (FPKM) were then calculated to represent the expression level of mRNA and lncRNA in each sample. The DE mRNAs and lncRNAs between the two groups were identified using DEseq. The DE cut-off criteria included *q* < 0.05 (adjusted *p* value) and |log_2_(Fold-change)| ≥ 1.0.

### circRNA Sequencing and Data Processing

The other cDNA library construction method was similar to that for the lncRNAs, except RNase R was used to remove linear RNAs. Clean reads were obtained by removing the following fragments: (1) low quality data, (2) reads containing an N ratio greater than 5%, and (3) reads containing jointed-sequence and rRNAs. BWA-MEM (Li, 2013) was used for mapping clean reads to the reference genome. circRNA Identifier (CIRI) (Gao et al., 2015), a highly efficient and fast circRNA recognition tool, was used for circRNA recognition. The BWA-MEM algorithm was used for sequence splitting, and then the resulting SAM file was scanned to detect paired chiastic clipping (PCC) and paired-end mapping (PEM) sites, as well as GT-AG splicing signals. Next, the sequence of junction sites was re-aligned using a dynamic programming algorithm to ensure the reliability of the identified circRNA. Spliced Reads per Billion Mapping (SRPBM) were used to determine the expression level of circRNA. The DE circRNAs between the two groups were identified using DEseq (Love et al., 2014). The cut-off criteria included *p* < 0.05 and |log_2_(Fold-change)| ≥ 1.0. circRNA–miRNA interactions were predicted using the miRanda (3.3a) prediction algorithm.

### miRNA Sequencing and Data Processing

18–30 nucleotide (nt) or 15–35 nt RNA fragments were obtained through agarose gel separation technology (Tao et al., 2019). Next, those RNAs were reverse transcribed to synthesize cDNA. Clean reads were obtained by removing the following fragments: (1) low quality data, (2) reads containing an N ratio greater than 10%, (3) reads without a 3’ linker sequence, (4) reads with polyA/T, and (5) reads without sequences. Bowtie1 (Langmead, 2010) was used to map the clean reads to the reference genome. Reads were mapped to mature miRNA and hairpin RNA that were recorded in miRBase (release 21) (Griffiths-Jones et al., 2008) to identify known miRNAs. After excluding the reads mapped to known miRNA/ncRNA/repeat regions/mRNA regions, the remaining reads were used to predict novel miRNAs based on their hairpin structure and stability. The miRDeep2 (Friedländer et al., 2011) software was applied for the identification and prediction of miRNAs. Transcripts per Million (TPM) was adopted to determine the expression levels of miRNAs. With a cut-off of *q* < 0.05 and | log_2_(Fold-change)| ≥ 1.0, the DESeq2 package in R software was employed to identify the DE miRNAs.

### Verification by Real-Time Quantitative Polymerase Chain Reaction

To verify the RNA-Seq results, 10 DE mRNAs were randomly selected for Real-time quantitative polymerase chain reaction (qRT-PCR) analysis (Table 1). Primer-BLAST of NCBI^2^ was used for primer design. Total RNA was reverse transcribed into cDNA using a PrimeScript RT Reagent Kit with gDNA Eraser according to the manufacturer’s instructions (RR047A, Takara, Japan). A 7500 Real-time PCR system (Applied Biosystems, CA, United States) was used to perform qRT-PCR. Actin was selected as the reference gene. The forward primer sequence was GATCTGGCACCACACCTTCT, and the reverse was GGGGTGTTGAAGGTCTCAAA. Three repeats were performed for each group. The relative gene expression levels were calculated by the 2^–ΔΔ*Ct*^ method (Livak and Schmittgen, 2001). qRT-PCR data were analyzed using a *t*-test with SPSS 13.0 software. A *p* value ≤0.05 was considered statistically significant.

**TABLE 1:** Primer sequences used in quantitative reverse-transcription polymerase chain reaction (RT-qPCR)-based verification of RNA sequencing results.

**Gene name**	**F-primer**	**R-primer**	**Log_2_ fold change(DUGL/AGL)**
Spp1	TCCACATTTCTGATGACCAGGAT	GGGCATGTTCAGACGATGGA	3.76
Col6a1	GCAGTCTTGGAAGGCAATAGG	CGAAGGCCAGCCAGAAACAT	0.3
Pla2g6	GTTGGCGCAGCTGATGATG	ATGCCCTGGTGAACTTCCAG	1.82
Pla2g4c	CTGCAAGTAGTGTAAGGGCT	GGGACAAATAAAGACTGCTGGA	–2.86
Nr4a3	TTTAACCCATGTCGCTCTGTGA	ATCGACTTCAGTGCCTTCGT	–3.45
Tfrc	TGAAAGTGGAATATCACTTCCTGTC	GCTAGGGCCAACTGGTTTCT	–1.2
Chrnb1	GTCCTCCTTCAGTGCGTCGT	CCTGAATTATCTGCCCCGGAC	–1.02
LOC100764246	AAGTCTCTCTCCATATCCTTCCTT	GTCCCAGTTAATGCAAAGCCC	–2.28
Mpp6	CCACCAAGCTTTTGACGGAC	CCCAGCTAATAGGGACCCAC	–1.35

*DUGL, deep underground lab; AGL, above ground lab.*

### Biological Information Analysis

Hierarchical clustering was conducted using R software (v3.5.1, 2018). Since the functional annotation of most lncRNAs has not been obtained, the functions of lncRNAs were predicted according to the annotations of the co-expressed mRNA function (Han et al., 2018). In this study, the function of DE lncRNAs were predicted based on position relationship (within 50 kb of lncRNA) and the Pearson correlation coefficient (the value of correlation ≥0.9, and *p* < 0.01) between lncRNA and mRNA.

The lncRNAs/circRNAs/miRNAs and mRNAs that shared expression levels with significant correlations were used to conduct co-expression analyses. To further reveal the biological functions of the DE RNAs, GO and KEGG pathway enrichment (performed using GeneCodis3 bioinformatics resources) were applied to the DE mRNAs, predicted targets for DE lncRNAs and miRNAs, and host genes of circRNAs. Both GO terms and KEGG pathways with *q* values < 0.05 were considered significantly enriched.

## Results

### Overview of Transcriptomic Analyses

Sequencing was performed on the cDNA and sRNA libraries of cell samples from the groups of DUGL and AGL cells grown for two days. Furthermore, total read counts and the ratio of mapped reads of the sequencing data are shown in Table 2.

**TABLE 2:** Summary of sequence after Illumina sequencing.

**Sample ID**	**circRNA**			**lnc + mRNA**			**miRNA**	
	**Total reads**	**Q30 (%)**	**Mapping Rate (%)**	**Total reads**	**Q30 (%)**	**Mapping Rate (%)**	**Total reads**	**Match Rate (%)**
V79_AGL-1	90517876	92.942	0.999	93212510	89.648	0.8496	10298183	60.14
V79_AGL-2	100037840	93.436	0.9992	70764158	90.731	0.8787	10525217	60.36
V79_AGL-3	97145502	93.31	0.9992	80015756	90.031	0.8671	10119350	60.45
V79_DUGL-1	103587538	92.925	0.9989	81711876	89.297	0.8584	10022116	64.5
V79_DUGL-2	92804208	92.918	0.9988	83057204	90.757	0.8761	10727785	67.31
V79_DUGL-3	82089486	92.828	0.9987	90595060	90.708	0.8612	10153452	64.28

*AGL, above ground lab; DUGL, deep underground lab.*

**FIGURE 1:**
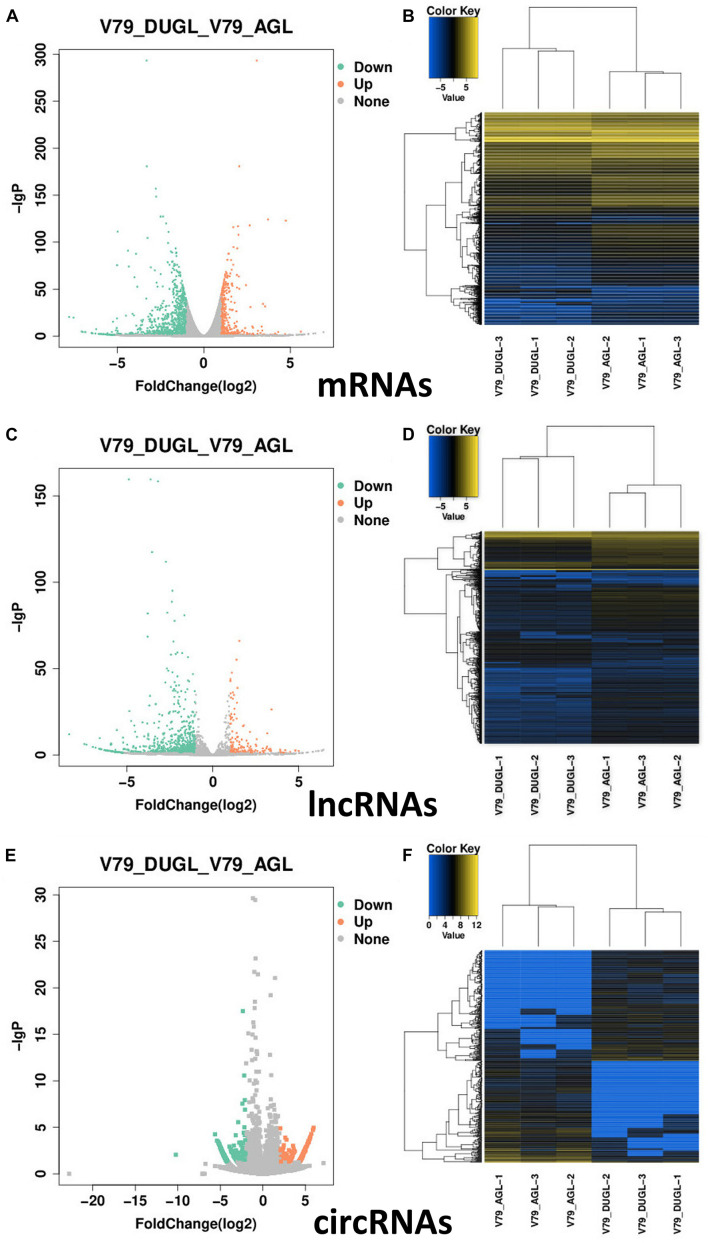
Summary of the differentially expressed (DE) RNAs between V79 cells cultured in either DUGL or AGL conditions. **(A)** Volcano plot of DE mRNAs. **(B)** Hierarchical cluster analysis of DE mRNAs. **(C)** Volcano plot of DE lncRNAs. **(D)** Hierarchical cluster analysis of DE lncRNAs. **(E)** Volcano plot of DE circRNAs. **(F)** Hierarchical cluster analysis of DE circRNAs. DUGL, deep underground laboratory; AGL, above ground laboratory. In the volcano plots, red and green dots correspond to RNAs that are significantly up-regulated or down-regulated between the two groups [—log_2_ fold change— ≥ 1.0 and *q* < 0.05 or (*p* < 0.05 for circRNAs)]. The *X*-axis shows the log_2_ fold changes of RNAs expression, and the *Y*-axis shows the adjusted *p*-value (−log_10_) for each gene. In the hierarchical cluster analyses, blue represents down-regulated, black represents no change, and yellow represents up-regulated.

Using a cut-off of | log_2_(Fold-change)| ≥ 1.0 and *q* < 0.05, a total of 1257 mRNAs and 866 lncRNAs were identified as being differentially expressed (DE) between the two groups. Using a | log_2_(Fold-change)| ≥ 1.0 and *p* < 0.05, 474 DE circRNAs were identified. However, only nine novel miRNAs were found to be down-regulated in DUGL cells, but these changes were not statistically significant. Among the DE RNAs, there were 353 up-regulated mRNAs, 904 down-regulated mRNAs, 145 up-regulated lncRNAs, 721 down-regulated lncRNAs, 247 up-regulated circRNAs, and 227 down-regulated circRNAs in the DUGL cells (Figure 1). The top 10 dysregulated lncRNAs, circRNAs and mRNAs are shown in Tables 3–5, respectively.

**TABLE 3:** The top ten differently expressed long non-coding RNAs (lncRNA) when comparing cells grown in either deep underground lab (DUGL) or above ground lab (AGL) conditions.

**Gene name**	**Log_2_ Fold Change**	***q* value**	**Location**
**lncRNAs(known)**			
LOC100758509_2	–5.66047	9.70E-04	chrNW_020822486.1:23460034-23522380:–
LOC103164300_1	–5.57904	3.51E-15	chrNW_020822370.1:33495091-33507253: +
LOC113832378	–5.21924	8.21E-11	chrNW_020822375.1:15663217-15671664: +
LOC103161061_1	–5.21393	6.48E-08	chrNW_020822504.1:18823253-18824765:–
LOC103161316_1	–5.12471	3.98E-04	chrNW_020822486.1:5308869-5315709:–
LOC113831664_1	–4.94432	0.00058	chrNW_020822438.1:1600081-1613566: +
LOC113832854	–4.92383	0.000751	chrNW_020822410.1:19442721-19456112:–
LOC113831251_1	–4.61109	0.007984	chrNW_020822467.1:6547657-6558607: +
LOC103159255_1	–4.50856	6.00E-03	chrNW_020822439.1:67883834-67884417: +
LOC113836383_1	–4.46703	0.000226	chrNW_020822486.1:23460034-23522380:–
LOC103159465_1	2.296029	0.002938	chrNW_020822423.1:371630-394726:–
LOC113835467_1	2.429808	0.01064	chrNW_020822530.1:8081052-8097556: +
LOC113833966_1	2.499678	8.84E-07	chrNW_020822455.1:921749-927513: +
LOC107978529_1	2.904763	0.004687	chrNW_020822428.1:4670842-4673304: +
LOC113831863_1	3.12391	0.000136	chrNW_020822603.1:3601354-3603135: +
LOC113831747_1	3.160932	1.45E-05	chrNW_020822438.1:1535637-1537003: +
LOC107978696_1	3.258981	0.000472	chrNW_020822455.1:931265-933279: +
LOC103163348_1	3.338026	0.003938	chrNW_020822439.1:14659910-14663129: +
LOC107979141_1	3.352202	0.000255	chrNW_020822398.1:20429447-20437433:–
LOC103159184_1	4.260327	0.049749	chrNW_020822530.1:9998432-10003516: +
**lncRNAs(novel)**			
MSTRG.59509	–6.57576	1.78E-10	chrNW_020822512.1:634352-636357: +
MSTRG.99600	–5.94264	1.22E-06	chrNW_020822619.1:2009350-2011797:–
MSTRG.21797	–5.93371	5.52E-07	chrNW_020822434.1:7402469-7414938: +
MSTRG.59508	–5.91585	2.43E-07	chrNW_020822512.1:618300-619177: +
MSTRG.44365	–5.89489	5.71E-07	chrNW_020822470.1:1277837-1279286: +
MSTRG.73727	–5.19106	0.000524	chrNW_020822559.1:236775-237739: +
MSTRG.47206	–5.17038	0.000402	chrNW_020822486.1:7934576-7936189: +
MSTRG.43863	–4.99667	0.000764	chrNW_020822468.1:45577419-45667919: +
MSTRG.50794	–4.98006	9.33E-07	chrNW_020822499.1:13398936-13400447:–
MSTRG.58014	–4.88975	0.002297	chrNW_020822507.1:14635785-14637831: +
MSTRG.91028	15.2715	0.006013	chrNW_020822608.1:5014765-5015598: +
MSTRG.112540	15.59807	0.03954	chrNW_020824056.1:4400-15732:–
MSTRG.68271	15.66354	0.034379	chrNW_020822532.1:98526-103809: +
MSTRG.59078	17.08523	0.0392	chrNW_020822511.1:1720903-1722420:–
MSTRG.83322	17.52892	0.048776	chrNW_020822601.1:1989261-1992807:–
MSTRG.104440	18.57067	0.001559	chrNW_020822644.1:302166-335565: +
MSTRG.110568	22.61577	0.017883	chrNW_020822701.1:6834752-6835186:–
MSTRG.91073	27.94233	0.002157	chrNW_020822608.1:5763552-5787063:–
MSTRG.47671	28.07458	0.002935	chrNW_020822486.1:21390271-21392110: +
MSTRG.62292	32.01071	0.005257	chrNW_020822520.1:3275391-3277354: +

**TABLE 4:** The top ten differently expressed circular RNAs (circRNA) when comparing cells grown in either deep underground lab (DUGL) or above ground lab (AGL) conditions.

**Genes name**	**Log_2_ Fold Change**	***p* value**	**Location**
cgr_circ_0012006	–10.2437	8.75E-03	NW_020822457.1:812975.834827:–
cgr_circ_0030911	–5.64245	5.56E-05	NW_020822601.1:43811847.43941220: +
cgr_circ_0015443	–5.39912	2.69E-04	NW_020822469.1:5198289.5321890:–
cgr_circ_0027306	–5.26727	3.62E-04	NW_020822567.1:60755438.60757648:–
cgr_circ_0009739	–5.23471	3.96E-04	NW_020822439.1:79249369.79251036:–
cgr_circ_0032228	–5.22167	0.000516	NW_020822604.1:2901429.2935648: +
cgr_circ_0007598	–5.15313	0.00061	NW_020822434.1:6416027.6455385: +
cgr_circ_0036359	–5.15101	0.000685	NW_020822616.1:2670879.2692762: +
cgr_circ_0016103	–5.11437	7.32E-04	NW_020822474.1:2563297.2567825: +
cgr_circ_0009519	–5.0494	0.001122	NW_020822439.1:73156436.73201595: +
cgr_circ_0024319	5.334614	0.000445	NW_020822532.1:11036157.11037777: +
cgr_circ_0014079	5.341966	0.000356	NW_020822465.1:12617038.12622922: +
cgr_circ_0004800	5.369736	0.000291	NW_020822412.1:6404901.6421685: +
cgr_circ_0003425	5.393138	0.000379	NW_020822406.1:21108493.21139948: +
cgr_circ_0000430	5.475466	0.000191	NW_020822370.1:36149042.36186242:–
cgr_circ_0036104	5.518788	0.000166	NW_020822615.1:7572164.7579808: +
cgr_circ_0005384	5.575185	0.000104	NW_020822415.1:41488127.41497361: +
cgr_circ_0036410	5.70205	5.18E-05	NW_020822616.1:3601937.3613766: +
cgr_circ_0038333	5.883023	1.92E-05	NW_020822643.1:1730572.1735016: +
cgr_circ_0009354	5.960931	1.1E-05	NW_020822439.1:61215838.61258669: +

**TABLE 5:** The top ten differently expression messenger RNAs (mRNA) when comparing cells grown in either deep underground lab (DUGL) or above ground lab (AGL) conditions.

**Genes name**	**Log_2_ Fold Change**	***q* value**	**Location**
**mRNA**			
LOC113835720	–7.79176	3.93E-21	chrNW_020822544.1:5811163-5811326: +
Nckap5	–7.53399	1.93E-20	chrNW_020822567.1:40287008-41073346: +
Slc28a1	–6.22702	4.23E-08	chrNW_020822504.1:18813688-18859764: +
LOC100769133	–5.5709	9.85E-04	chrNW_020822526.1:24986219-25027768:–
Ccl20	–5.32189	4.00E-05	chrNW_020822439.1:55494743-55497949: +
Sema4d	–5.24503	5.26E-16	chrNW_020822507.1:12095408-12200001: +
LOC103160046	–5.08814	7.44E-07	chrNW_020822420.1:11663167-11663819:–
Adgra2	–5.03528	2.58E-09	chrNW_020822436.1:4972248-5013378: +
Mmp9	–5.01822	1.27E-19	chrNW_020822603.1:8682735-8691181:–
Arhgap36	–5.01231	1.74E-76	chrNW_020822701.1:887109-894443:–
LOC113835065	3.538876	2.37E-32	chrNW_020822507.1:14787594-14790149:–
LOC113838100	3.71766	5.6E-125	chrNW_020823296.1:2190-13207:–
Ptgdr2	3.860848	0.049366	chrNW_020822499.1:25125224-25128118:–
LOC100763630	3.972035	0.040251	chrNW_020822381.1:901091-903572: +
Myo7b	4.113255	0.001768	chrNW_020822458.1:11022595-11094756:–
Trnag-ccc	4.222555	0.044225	chrNW_020822423.1:1750970-1751040: +
Adcy3	4.298111	3.85E-05	chrNW_020822608.1:5019188-5096634: +
LOC113837896	4.699751	0.009909	chrNW_020822789.1:7-21637:–
Nr4a3	4.743004	7.7E-124	chrNW_020822468.1:11614911-11657680:–
Cd160	5.612465	1.72E-05	chrNW_020822423.1:2286254-2301351:–

Hierarchical clustering of the lncRNA, mRNA and circRNA expression suggested obvious discrimination in V79 cells between the DUGL and AGL growth conditions (Figure 1).

### The Functional Analysis of DE mRNAs

**FIGURE 2:**
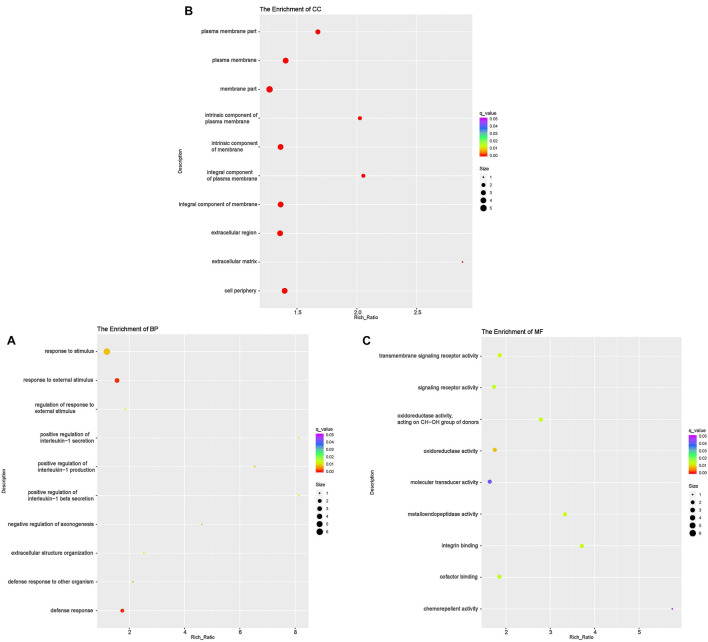
Functional annotation of the differentially expressed (DE) down-regulated RNAs messenger (mRNA) with the top 10 rich ration of biological processes (BP) **(A)**, cellular component (CC) **(B)**, and molecular function (MF) **(C)**. The abscissa is the rich ration [(DE genes of the term/all the DE genes)/(all the annotated gene of the terms/all the annotated gene)]; the *Y*-axis shows the terms enriched. A higher rich ration correlates with lower *p*-values. Circle size represents the number of enriched genes, and the color indicates the degree of enrichment, with red representing the highest degree of enrichment.

**FIGURE 3:**
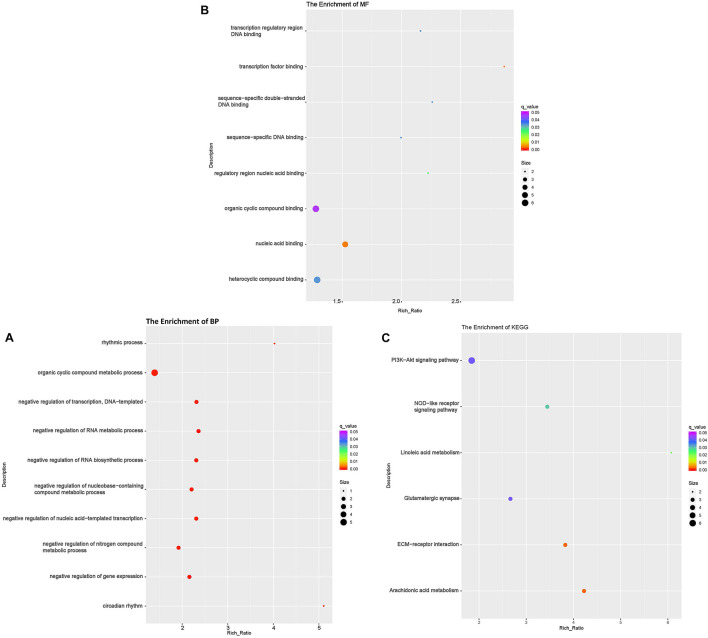
Functional annotation of the differentially expressed (DE) up-regulated RNAs messenger (mRNA) with the top 10 rich ration of biological processes (BP) **(A)**, molecular function (MF) **(B)**, and KEGG pathways **(C)**. The abscissa is the rich ration [(DE genes of the term or pathway/all the DE genes)/(all the annotated gene of the term or pathway/all the annotated gene)]; the *Y*-axis shows the terms enriched. A higher rich ration correlates with lower *p*-values. Circle size represents the number of enriched genes, and the color indicates the degree of enrichment, with red representing the highest degree of enrichment.

According to GO analyses, in the biological process (BP) category, the down-regulated mRNAs in the DUGL group were enriched in 28 terms (Table 6). The top three enriched terms were response to external stimulus(GO:0009605), defense response(GO:0006952) and response to stimulus(GO:0050896) (Figure 2A and Supplementary Table 1). The up-regulated mRNAs in the DUGL group were enriched in 102 BP terms (Table 6, Figure 3A and Supplementary Table 2). Among those BP terms, 19 terms were negative regulation terms, which covered gene and metabolic processes. Moreover, seven negative regulation terms ranked in the top 10 enriched terms (Figure 3A and Supplementary Table 2). In the cellular component (CC) category, the down-regulated mRNAs were mainly enriched in terms of membrane part [e.g., plasma membrane (GO:0005886), extracellular region part (GO:0044421) and extracellular space(GO:0005615) (Figure 2B and Supplementary Table 1)]; whereas the up-regulated mRNAs were only enriched in terms of the nucleolus (GO:0005730) (Supplementary Table 2). As to the molecular function (MF) category, the down-regulated mRNAs were mainly enriched in terms of oxidoreductase activity (GO:0016614), transmembrane signaling receptor activity (GO:0004888) and metalloendopeptidase activity(GO:0004222) (Figure 2C and Supplementary Table 1); whereas the up-regulated mRNAs were mainly enriched in terms of binding [e.g., transcription factor(GO:0008134),nucleic acid(GO:0003676) and regulatory region nucleic acid(GO:0001067) (Figure 3B and Supplementary Table 2)].

**TABLE 6:** The overview of functional analysis of differentially expressed RNAs.

**RNAs**	**GO(term)**			**KEGG (pathway)**
	**Biological process**	**Cellular component**	**Molecular function**	
**mRNAs**				
Down-regulated	28	27	9	6
Up-regulated	102	1	8	
**lnc RNAs**				
Down-regulated	173	85	39	4
Up-regulated	214	101	53	3
**circRNAs**				
Down-regulated	4	8	9	
Up-regulated		31	3	1

*GO, Gene Ontology; KEGG, Kyoto Encyclopedia of Genes and Genomes.*

The KEGG analysis showed that the down-regulated mRNAs were enriched in six pathways including extracellular matrix-receptor interaction (ECM-RI), arachidonic acid metabolism, linoleic acid metabolism, NOD-like receptor signaling pathway, glutamatergic synapse and PI3 kinase-Akt signaling pathway (Figure 3C and Supplementary Table 3). There was no significant enrichment for up-regulated mRNAs in any pathway.

### The Functional Analysis of DE lncRNAs

**FIGURE 4:**
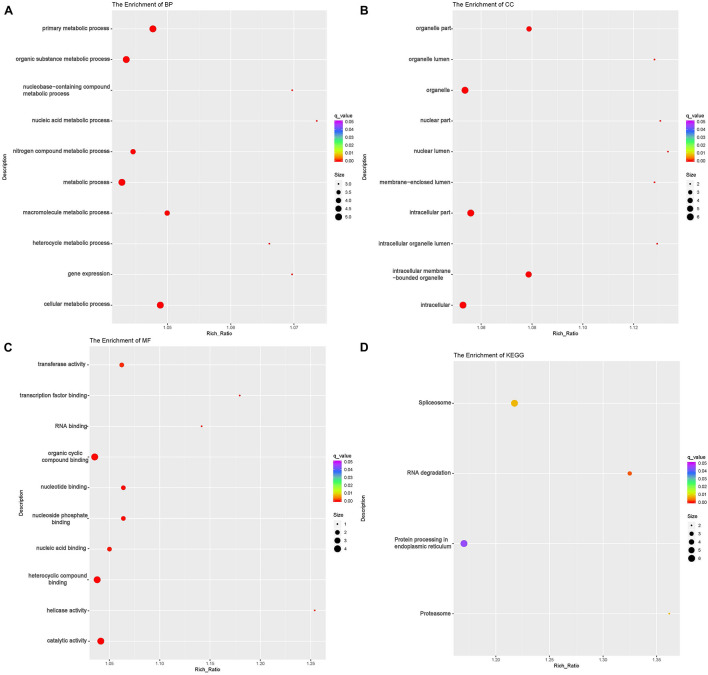
Functional annotation of predicted targets of differentially expressed (DE) down-regulated long non-coding RNAs (lncRNA) with the top 10 enrichment scores of biological processes (BP) **(A)**, cellular component (CC) **(B)**, molecular function (MF) **(C)**, and KEGG pathways **(D)**. The abscissa is the rich ration [(DE genes of the term or pathway/all the DE genes)/(all the annotated gene of the term or pathway/all the annotated gene)]; the *Y*-axis shows the terms enriched. A higher rich ration correlates with lower *p*-values. Circle size represents the number of enriched genes, and the color indicates the degree of enrichment, with red representing the highest degree of enrichment.

**FIGURE 5:**
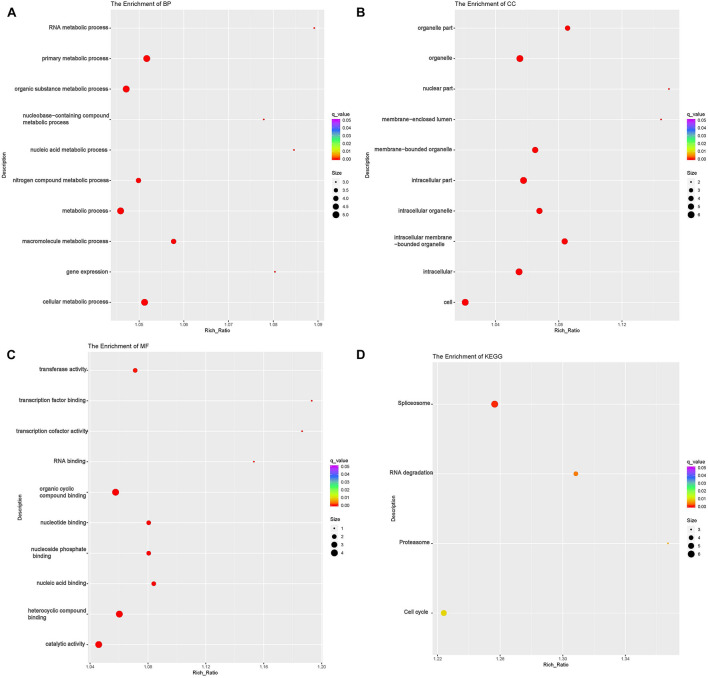
Functional annotation of predicted targets of differentially expressed (DE) up-regulated long non-coding RNAs (lncRNA) with the top 10 enrichment scores of biological processes (BP) **(A)**, cellular component (CC) **(B)**, molecular function (MF) **(C)**, and KEGG pathways **(D)**. The abscissa is the rich ration [(DE genes of the term or pathway/all the DE genes)/(all the annotated gene of the term or pathway/all the annotated gene)]; the *Y*-axis shows the terms enriched. A higher rich ration correlates with lower *p*-values. Circle size represents the number of enriched genes, and the color indicates the degree of enrichment, with red representing the highest degree of enrichment.

To investigate the DE lncRNAs under the LBR stress of the deep underground environment, a functional analysis was performed for the predicted target mRNA of the DE lncRNAs (145 up-regulated lncRNAs, 721 down-regulated lncRNAs; Figures 1C,D). Of those DE lncRNA, 497 lncRNAs were novel lncRNAs. The predicted target mRNAs of down-regulated lncRNAs were mainly enriched in 173 BP terms, 85 CC terms and 39 MF terms (Table 6 and Supplementary Table 4). The BP terms were mainly related to several steps of the metabolic process (Figure 4A), the CC terms were related to organelle (Figure 4B) and the MF terms were related to binding (Figure 4C). In contrast, the predicted target mRNAs of the up-regulated lncRNAs were mainly enriched in 214 BP terms, 101 CC terms and 53 MF terms (Table 6 and Supplementary Table 5). In the BP category, the main enriched terms were related to metabolic processes (GO:0008152), such as primary (GO:0044238), organic substance(GO:0071704) and macromolecule metabolic (GO:0043170) (Figure 5A). Part of those terms were enriched in the cellular response to stress (GO:0033554), and regulation of cellular response to stress (GO:0080135) (Supplementary Table 5). In the CC category, the target mRNAs of DE lncRNAs mainly related to organelle part (GO:0044422), organelle (GO:0043226), and nuclear part (GO:0044428) (Figure 5B). As for the MF category, most of the identified terms related to binding (GO:0005488), catalytic (GO:0003824), and protein binding (GO:0005515) functions (Figure 5C).

In the KEGG analysis, the target mRNAs of down-regulated lncRNAs were significantly enriched in four pathways (spliceosome, RNA degradation, proteasome and protein processing in the endoplasmic reticulum) (Figure 4D). Interestingly, the target mRNAs of up-regulated lncRNAs were enriched in these same three pathways and one other pathway (spliceosome, RNA degradation, proteasome and cell cycle) (Figure 5D).

### The Functional Analysis of DE circRNAs

**FIGURE 6:**
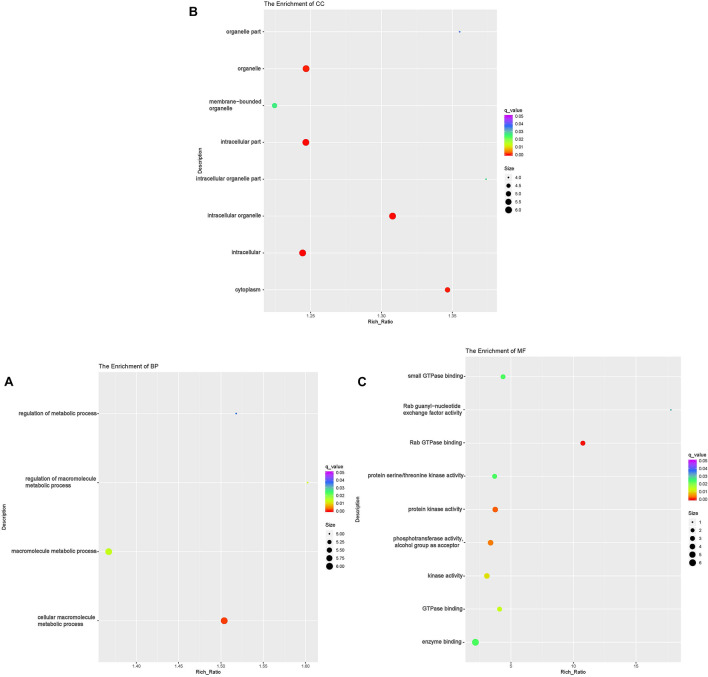
Functional annotation of host genes of differentially expressed (DE) down-regulated circular RNAs (circRNA) with the top 10 rich ration of biological processes (BP) **(A)**, cellular component (CC) **(B)**, and molecular function (MF) **(C)**. The abscissa is the rich ration. The abscissa is the rich ration [(DE genes of the term/all the DE genes)/(all the annotated gene of the term / all the annotated gene)]; the *Y*-axis shows the terms enriched. A higher rich ration correlates with lower *p*-values. Circle size represents the number of enriched genes, and the color indicates the degree of enrichment, with red representing the highest degree of enrichment.

**FIGURE 7:**
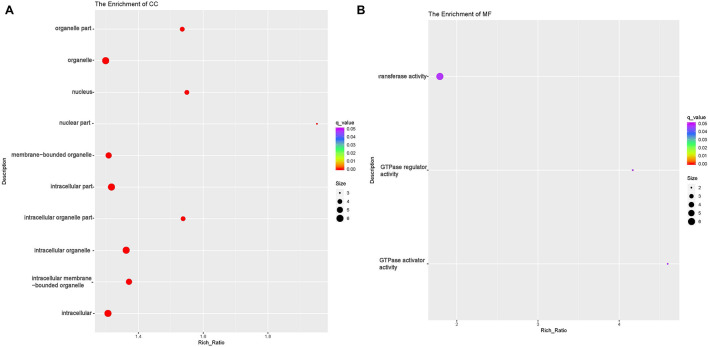
Functional annotation of host genes of differentially expressed (DE) up-regulated circular RNAs (circRNA) with the top 10 rich ration of cellular component (CC) **(A)** and molecular function (MF) **(B)**. The abscissa is the rich ration [(DE genes of the term /all the DE genes)/(all the annotated gene of the term / all the annotated gene)]; the *Y*-axis shows the terms enriched. A higher rich ration correlates with lower *p*-values. Circle size represents the number of enriched genes, and the color indicates the degree of enrichment, with red representing the highest degree of enrichment.

circRNAs exert their functions through host genes, and 474 DE circRNAs (247 up-regulated, 227 down-regulated) were detected in this study. Function analyses were then performed to identify the host genes of these DE circRNAs. GO analyses showed that host genes of the down-regulated circRNAs were enriched in four BP terms [(cellular macromolecule metabolic process (GO:0044260), regulation of macromolecule metabolic process (GO:0060255), macromolecule metabolic process (GO:0043170) and regulation of metabolic process (GO:0019222)];eight CC terms including intracellular part (GO:0044424) and organelle (GO:0043229) and nine MF terms including Rab GTPase binding(GO:0017137), protein kinase activity(GO:0004672) and phosphotransferase activity(GO:0016773) (Table 6 and Figures 6A–C, and Supplementary Table 6). The host genes of the up-regulated circRNA were enriched in 31 CC terms including intracellular part(GO:0044424) and organelle(GO:0043229);and three MF terms [GTPase activator(GO:0005096) and regulator activity(GO:0030695), transferase activity(GO:0016740)] (Table 6 and Figures 7A,B). Several terms surrounding metabolic processes were enriched, which was similar to the target mRNAs of the DE lncRNAs. Kyoto Encyclopedia of Genes and Genomes (KEGG) pathway analyses showed that the host genes of down-regulated circRNA were enriched in protein processing in the endoplasmic reticulum.

### Construction of the circRNA-miRNA Co-expression Network

**FIGURE 8:**
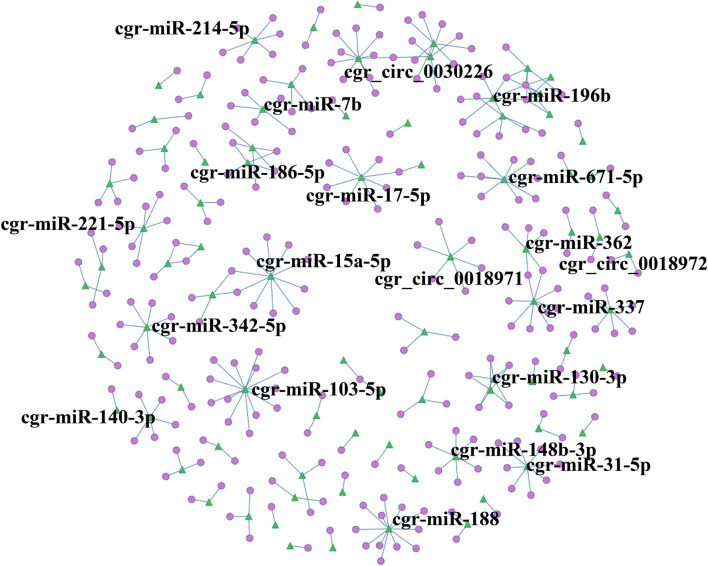
circRNA-miRNA co-expression network based on the RNA sequencing results. Circles represent circRNAs, and green triangles represent miRNAs. The label was the name of RNA.

A circRNA-miRNA co-expression network was constructed based on the RNA-Seq results, and when comparing DUGL to AGL cells, 286 miRNA-circRNA interaction pairs were obtained. For interest, the miRNA-circRNA interaction network was shown in Figure 8 and Supplementary Table 8.

### Verification of DE RNA by qRT-PCR

**FIGURE 9:**
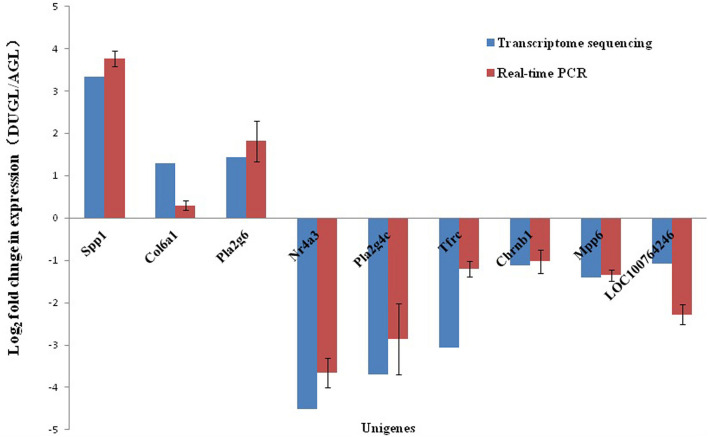
Expression relationship of nine differentially expressed genes validated by qRT-PCR and RNA sequencing.

To verify the RNA-Seq results, 10 DE mRNAs were selected for qRT-PCR analysis. Among them, nine mRNAs had comparable expression patterns between the RNA-Seq and qRT-PCR results (Figure 9).

## Discussion

Several researchers have found that the deep underground environment, where cosmic radiation is shielded, can reduce the growth rates of paramecia, bacteria and some mammalian cells (Planel et al., 1987; Smith et al., 2011; Castillo et al., 2015). Indeed, cell translational repression and gene expression profiles induced by stress can inhibit proliferation (Wang et al., 2015). However, as an environmental stress, little is known about the genetic profile changes that occur under the stress of LBR in a deep underground environment. In this study, whole transcriptomic analyses were conducted in V79 cells grown for two days in a DUGL with LBR and compared to cells grown in an AGL. The results showed a distinct genetic profile change, which covered circRNAs, mRNAs, and lncRNAs (most of them down-regulated). Although there was no significant difference in miRNA between the two groups, nine DE novel miRNAs were identified. Ten DE mRNAs found by RNA-Seq were selected for qRT-PCR validation, and a similar expression level in nine of the ten mRNAs confirmed the accuracy of the RNA-seq findings to some extent. Taken together, the changes in the gene profile suggested that LBR stress could cause a delay in growth through the inhibition gene transcription. Therefore, the genetic down-regulation induced by LBR stress might be the main molecular basis of the inhibition of proliferation in V79 cells cultured in DUGL.

The GO term annotation is helpful to reveal physiological and functional changes related to genes and protein expression in cells (Camon et al., 2004). To further explore the effect of the genetic changes in V79 cells cultured in DUGL, GO analyses were performed for the DE mRNAs. Under LBR environmental stress, the top three BP terms of the down-regulated mRNAs were related to responses to stimulus and defense, and the down-regulated mRNA mainly enriched in CC terms of plasma membrane. The cell senses and adapts to changes in the extracellular environment by plasma membrane, which allows the external singal inputing via intracellular signaling networks (Rausch and Hansen, 2020). The down-regulated mRNAs of plasma membrane related to stress function might help to explain the hypothesis that normal environmental radiation contributes to maintaining the defense systems of living organisms (Fratini et al., 2015). Contrast to γ irradiation could induce the expression of the interleukin-1(IL-1) gene (Bigildeev et al., 2013), our study showed that the LBR could decrease the IL-1 gene production, which presented with the down-regulate mRNAs enriched in ten GO terms involving IL-1 production. On the other hand, the up-regulated DE mRNAs were significantly enriched in BP categories involved in negative regulation terms, such as gene expression, metabolic process, and biosynthetic process. These up-regulated mRNAs related to negative metabolic and biosynthetic functions also could be the main causative factors of proliferative inhibition.

The KEGG pathway analysis is useful for the systematic understanding of large-scale gene functions. In this study, KEGG pathway analysis of DE mRNAs showed significant enrichment in ECM-RI, arachidonic acid metabolism, linoleic acid metabolism, NOD-like receptor signaling pathway, glutamatergic synapse and PI3K-Akt signaling pathway. As a biological regulation network, both the ECM-RI and PI3K-Akt signaling pathways were comprehensive net and played an essential role in cell proliferation and survival (Landgren and Curtis, 2011; Zhang G. et al., 2018). Twelve down-regulated were mRNAs shared in the two pathways. Most of the down-regulated genes had the function of promoting proliferation, such as collagen alpha 1 (Col1a1) (Jin et al., 2017), integrin alpha 7 (Itga7) (Ge et al., 2020), laminin beta 3 (Lamb3) (Wang et al., 2018) and secreted phosphoprotein 1 (Spp1) (Zeng et al., 2018). Furthermore, other down-regulated genes were detected with similar functions [e.g., Rab40b (Jacob et al., 2016), S100 calcium-binding protein A4 (S100A4) (Forman et al., 2010) and collagen prolyl-4-hydroxylase α subunit 2 (P4HA2)]. These key down-regulated genes might play crucial roles in the inhibition of growth found in DUGL cultures. Moreover, arachidonic and linoleic acid metabolism, as well as NOD-like receptor signaling and the glutamatergic synapse might also be involved in the stress response of LBR. However, these pathways’ role in LBR stress need further research.

Besides the DE mRNAs enriched in GO terms and KEGG pathways, several top DE mRNAs function in the regulation of cell proliferation. Matrix metallopeptidase 9 (MMP9) has been shown to be involved in promoting proliferation (Lan et al., 2020). Human concentrative nucleoside transporter-1 (hCNT1, SLC28A1) (Wang and Buolamwini, 2019), Ccl20 (Qiu et al., 2011), Sema4d (Bhutia et al., 2011), and Arhgap36 (Rack et al., 2014) have been shown to influence cellular growth and proliferation, and were the top down-regulated mRNAs in V79 cells cultured in a DUGL. Nuclear receptor 4A3 (NR4A3) has been shown to have the ability to suppress cell growth (Zhang B. et al., 2018; Parsa et al., 2019). Importantly, NR4A3 was significantly up-regulated in cells cultured in a DUGL. Therefore, it can be inferred that the down-regulation of genes that promote growth and the up-regulation of genes that function to suppress growth also contribute to the inhibition of proliferation in cells cultured in a DUGL.

Non-coding RNAs, by definition, do not code for protein. However, they have crucial roles in various cellular activities (Kukleva et al., 2015). Therefore, the lncRNA, circRNA, and miRNA in V79 cells with altered expression profiles between those cultured in a DUGL and AGL were comprehensively investigated. lncRNA is a class of non-coding transcripts longer than 200 nucleotides (Cao et al., 2018). In the present study, 866 lncRNAs were found to be differentially expressed between the two groups. The number of down-regulated lncRNAs was much higher than those that were up-regulated, suggesting that the expression of lncRNAs transcripts is repressed by LBR stress. Owing to a few studies in this field, more than half of the DE lncRNAs were newly identified in this study.

To further reveal the functions of the identified DE lncRNAs, GO and KEGG analyses were performed. In the BP category, both up- and down- regulated target mRNAs of lncRNAs were mainly enriched in many terms related to metabolic processes. This finding strongly suggested that lncRNAs, similar to mRNAs, might also play a crucial role by altering metabolic processes during LBR stress in a deep underground environment. In the KEGG analyses, the target mRNAs of dysregulated lncRNAs shared three pathways including spliceosome, RNA degradation and proteasome. Spliceosomes are known to precisely and efficiently perform mRNA processing, which is a critical step in organ development. Consistent with proteomic result of our previous research (Liu et al., 2020), the target mRNAs of lncRNAs enriched in these pathways indicated that the spliceosome played an important role in the LBR stress response of V79 cells. Additionally, the involvement of the RNA degradation and proteasome pathways might suggest that these pathways also function in the stress response in a LBR environment.

Regarding another class of non-coding RNA, accumulating evidence has highlighted that circRNAs can affect mRNA splicing and transcription (Zhang et al., 2019). Therefore, we analyzed the DE circRNAs between DUGL and AGL groups of V79 cells and identified 474 DE circRNAs. GO analyses showed that the host genes of down-regulated DE circRNAs were enriched in metabolic processes, which was similar to the results found in both DE mRNAs and target genes of DE lncRNAs. This finding indicated that circRNAs are also involved in the LBR stress of V79 cells by interacting with lncRNAs and mRNAs. The endoplasmic reticulum (ER) is a vital organelle that can perceive environmental changes (Wang et al., 2019). ER stress could induce changes in key mediators for cell survival (Chien et al., 2017). KEGG analyses revealed that the host genes of down-regulated DE circRNAs were enriched in the pathway of protein processing in the ER. This result was consistent with our previous studies, which have revealed that several proteins of the ER were down-regulated in cells cultured in a DUGL. However, further confirmation is required to verify the functional role of the ER in LBR stress.

miRNAs, as a class of small non-coding RNAs (approximately 22 nucleotides), are essential elements to regulate gene expressions through partial base-pairing with target mRNAs (Lin et al., 2018). circRNAs may function similarly to regulate the activity of other miRNAs (Wilusz and Sharp, 2013). Due to little research in this area, and since the minority of the miRNA functions were annotated, we failed to detect significance difference in the DE miRNAs between the two groups. However, we identified several circRNAs that contained one or more miRNA binding sites and obtained 286 miRNA-circRNA interaction pairs between the DUGL and AGL groups. These interactions and their functions involved in LBR stress are worthy of being investigated further.

Nucleic acid binding plays an important role in translation regulation (Liu et al., 2020). In our present study, the up-regulated mRNA and target mRNAs of dysregulated lncRNAs enriched in many GO terms which were related to nucleic acid binding. The change of gene expression was consistent with the proteomic result of V 79 cells conducted in our previous research (Liu et al., 2020). Those molecular change from RNAs to proteins further indicated that these nucleic acid binding was affected when V 79 cells under the stress of reduced background radiation.

Environmental stress can trigger an increase in reactive oxygen species (Fan et al., 2015). Castillo and colleagues (Castillo et al., 2015) have shown that oxidative stress and the SOS response (*katB* and *recA*) as well as metal efflux activity (*SOA0154*) were elevated in cells grow under LBR conditions. Although we did not detect the differential expression of these specific genes between the two groups, down-regulated DE mRNAs in DUGL cells were found to be enriched in two terms involving oxidoreductase activity. Similar to DE RNAs, the predicted target genes of DE lncRNAs were observed to be enriched in cellular responses to oxygen-containing compounds and mitochondrial terms. These findings were also consistent with our previous research, which showed that cells grown in a DUGL presented a change in energy metabolism, morphologic changes of mitochondrion and oxidative phosphorylation (Liu et al., 2020). Taken together, the results of this study suggest that the oxidative response could be involved in the LBR stress response in the deep underground environment.

The limitations of our study included the short growth time of V79 cultured cells (two days) in the DUGL for the analysis of lncRNAs, circRNAs, and miRNA. Also, we did not verify the sequencing results for DE lncRNAs and circRNAs by RT-qPCR. Additionally, we failed to construct a competing endogenous network and verify an interactive relationship. Therefore, additional in-depth research is required to reveal the biological specifics of the LBR stress response in a deep underground environment.

## Conclusion

In conclusion, our study investigated the transcription patterns of lncRNAs, mRNAs, circRNAs, and miRNAs of V79 cells cultured in a DUGL and an AGL by whole-transcriptome sequencing and integrated analysis. We confirmed that the LBR of a deep underground environment could induce V79 cell transcription repression, metabolic process delaying and overall gene expression profile reprogramming. The altered RNA profiles were mainly discovered in lncRNAs, mRNAs and circRNAs. DE RNAs were involved in many pathways including ECM-RI, PI3K-Akt signaling, RNA transport and the cell cycle under the LBR stress of the deep underground environment. These profile changes might be the molecular basis of the inhibition of cell proliferation. This study provided a systematic perspective on the potential effects of the deep underground environment on V79 cells.

## Data Availability Statement

The datasets generated and/or analyzed during the current study can be found with this article and Supplementary Information online. The raw transcriptomic data have been deposited to the NCBI Short Read Archive (SRA) under the BioProject accession number PRJNA623056: https://www.ncbi.nlm.nih.gov/bioproject/PRJNA623056.

## Author Contributions

JL, LD, SL, JZ, JW, HX, and WL conceived and designed the experiments. JL, LD, YX, JC, and LW conducted the experiments. JL, HJ, YL, LD, TM, and MG analyzed the data. JL and LD wrote the manuscript. SL, HJ, and JZ revised the manuscript. All authors read and approved the final version of this manuscript.

## Conflict of Interest

The authors declare that the research was conducted in the absence of any commercial or financial relationships that could be construed as a potential conflict of interest.

## Publisher’s Note

All claims expressed in this article are solely those of the authors and do not necessarily represent those of their affiliated organizations, or those of the publisher, the editors and the reviewers. Any product that may be evaluated in this article, or claim that may be made by its manufacturer, is not guaranteed or endorsed by the publisher.

## Footnotes

1
http://ccb.jhu.edu/software/hisat2/index.shtml

2
https://www.ncbi.nlm.nih.gov/tools/primer-blast/index.cgi
